# The EMT spectrum and therapeutic opportunities

**DOI:** 10.1002/1878-0261.12082

**Published:** 2017-06-19

**Authors:** Dominic C. Voon, Ruby Y. Huang, Rebecca A. Jackson, Jean P. Thiery

**Affiliations:** ^1^ Institute for Frontier Science Initiative Kanazawa University Ishikawa Japan; ^2^ Division of Genetics Cancer Research Institute Kanazawa University Ishikawa Japan; ^3^ Department of Obstetrics & Gynaecology National University Hospital Singapore Singapore; ^4^ Cancer Science Institute of Singapore National University of Singapore Singapore Singapore; ^5^ Department of Biochemistry Yong Loo Lin School of Medicine National University of Singapore Singapore Singapore; ^6^ Inserm Unit 1186 Comprehensive Cancer Center Institut Gustave Roussy Villejuif France; ^7^ CNRS UMR 7057 Matter and Complex Systems University Paris Denis Diderot Paris France

**Keywords:** cancer therapeutics, cellular plasticity, drug discovery, drug resistance, EMT spectrum, epithelial‐mesenchymal transition

## Abstract

Carcinomas are phenotypically arrayed along an epithelial–mesenchymal transition (EMT) spectrum, a developmental program currently exploited to understand the acquisition of drug resistance through a re‐routing of growth factor signaling. This review collates the current approaches employed in developing therapeutics against cancer‐associated EMT, and provides an assessment of their respective strengths and drawbacks. We reflect on the close relationship between EMT and chemoresistance against current targeted therapeutics, with a special focus on the epigenetic mechanisms that link these processes. This prompts the hypothesis that carcinoma‐associated EMT shares a common epigenetic pathway to cellular plasticity as somatic cell reprogramming during tissue repair and regeneration. Indeed, their striking resemblance suggests that EMT in carcinoma is a pathological adaptation of an intrinsic program of cellular plasticity that is crucial to tissue homeostasis. We thus propose a revised approach that targets the epigenetic mechanisms underlying pathogenic EMT to arrest cellular plasticity regardless of upstream cancer‐driving mutations.

**Abbreviations**
CSCs: cancer stem cells
CTCs: circulating tumor cells
EMP: epithelial–mesenchymal plasticity
EMT: epithelial–mesenchymal transition
HCC: hepatocellular carcinoma
HDAC: histone deacetylases
HDACi: histone deacetylase inhibitor
MET: mesenchymal‐epithelial transition
NSCLC: non‐small cell lung cancer
PHF2: PHD finger protein 2
PKA: protein kinase A
TET1: ten‐eleven translocation 1

## 1 The EMT spectrum

Recent evidence has advanced and broadened the definition of epithelial–mesenchymal transition (EMT) in human pathologies. While earlier studies relied on the use of key epithelial and mesenchymal markers to detect its aberrant activation during pathogenesis, it now becomes clear that this is a not a simple binary decision to acquire either an epithelial or a mesenchymal state. Rather, pathological EMT manifests dynamic transitional states punctuated by metastable intermediates (Nieto *et al*., 2016). This review collates the current knowledge of the molecular mechanisms underlying this phenomenon, and discusses current efforts in the deployment and development of therapeutic interventions.

EMT is orchestrated by a core set of transcription factors (EMT‐TFs), each having the ability to drive EMT via largely analogous genetic programs. These include SNAI1/2, TWIST, and ZEB, among others. As reviewed elsewhere, a myriad of growth factor and developmental signals activate these EMT‐TFs (Thiery *et al*., 2009). However, the precise reasons for why this highly controlled program is aberrantly triggered at times are varied and often obscured. This is compounded by the inherent difficulty in quantifying the extent of the so‐called partial EMT in each disease state – just exactly how stable is metastable? Such complexities present a formidable challenge in rational drug design. Indeed, with such variations, what works in one context or in a particular patient could be futile or harmful in another. Nevertheless, with fresh knowledge and the benefit of hindsight, certain principles have emerged.

Like with other examples of heterogeneity encountered in biology, there is also heterogeneity following the execution of the EMT program. One explanation is that EMT heterogeneity results from a diverse mix of populations undergoing EMT at different rates and downstream to various cues. For example, circulating tumor cells (CTCs) isolated from patients with breast cancer display a spectrum of epithelial–mesenchymal hybrid features (Khoo *et al*., 2015; Yadavalli *et al*., 2017; Yu *et al*., 2013a), the composition of which varies significantly among patients and is greatly dominated by the underlying biology of the primary tumor. Along the clinical course, the epithelial–mesenchymal hybrid features of CTCs continue to evolve, further illustrating that the metastable state itself exists as a dynamic range of equilibrium. With this appreciation of EMT as a spectrum of different states, broader perspectives of how to manipulate the metastable state within each context can thus be provided.

## 2 EMT drug discovery platforms

At the heart of each drug discovery platform is a cohesive concept. In the development of EMT‐targeting therapeutics, the following approaches have been adopted: (a) killing cells that have undergone EMT and (b) reversing EMT in metastable cells. It is worth noting here that while these approaches share a common purpose, the rationale for each is distinct.

### 2.1 Targeting EMT‐induced cancer stem cells

In addition to greater chemoresistance, cells that have undergone EMT bear increased stem‐like traits *in vitro* (Mani *et al*., 2008; Morel *et al*., 2008) and *in vivo* (Guo *et al*., 2012); this observation raised the hope that targeting EMT could eradicate the rare self‐renewing and multipotent ‘cancer stem cells’ (CSCs) that persist following conventional chemotherapy. EMT is also associated with increased cell migration and resistance to anoikis, properties that are associated with tumor invasion and metastasis. Thus, the specific killing of cells that have undergone EMT is an attractive therapeutic strategy against CSCs.

To date, the most extensive and prominent EMT‐targeting screen was performed on the HMLE series of immortalized human mammary epithelial lines. These lines have been well characterized in studies of cellular transformation (Elenbaas *et al*., 2001). This model system led to the discovery of the EMT‐induced, tumor‐initiating CSC, typified by their CD44^high^/CD24^low^ phenotype (Mani *et al*., 2008; Morel *et al*., 2008). The production of these cells was shown to be achieved either through the forced expression of EMT‐TFs (SNAI1, TWIST1, and ZEB1) or through a combination of growth factors and RNAi (shEcad) (Mani *et al*., 2008; Scheel *et al*., 2011).

A high‐throughput screen in a 384‐well format was conducted using an HMLE derivative line that was induced to undergo EMT by expressing shEcad. This screen identified the selective cytotoxic effects of salinomycin, a potassium ionophore hitherto known as an antibiotic, on the CSC subpopulation >100‐fold relative to paclitaxel (Gupta *et al*., 2009). Subsequent studies revealed that salinomycin promotes the degradation of the Wnt coreceptor LRP6 (lipoprotein receptor‐related protein 6) by inhibiting its phosphorylation, thereby attenuating Wnt signaling (Lu *et al*., 2011). The HMLE platform was further deployed in expanded screens identifying other candidate compounds, most notably ML239, which appears to target NF‐κB signaling (Carmody *et al*., 2012). More recently, a synthetic derivative of salinomycin was shown to kill breast CSCs by sequestering iron in the lysosome, thereby triggering ferroptosis (Mai *et al*., 2017).

However, despite these advances, there are potential drawbacks to the cytotoxic killing of carcinoma cells undergoing an EMT. First, the endpoint of their transition is often not a permanent mesenchymal state but rather a metastable intermediate state, thus rendering them difficult to target. Indeed, the spectrum of intermediate states exhibited by CTCs (Khoo *et al*., 2015; Yadavalli *et al*., 2017; Yu *et al*., 2013a) likely means that they are not an effective target. Second, cytotoxicity exerts a selective pressure that may hasten the evolution of CSCs into alternative metastable states not sensitive to the drug.

### 2.2 Reversing EMT in metastable cancer cells

In using an EMT reversal approach, mesenchymal‐like carcinoma cells are reverted to their epithelial‐like (original) phenotype, thereby restricting the (acquired) self‐renewal and invasive properties of these cancer cells. However, few suitable models exist for testing noncytotoxic, EMT‐reversing agents. One platform used the NBT‐II rat bladder carcinoma line to screen for compounds that could reverse growth factor‐induced cell scattering (Chua *et al*., 2012). Although modest in scale, this screen identified noncytotoxic compounds that target ALK5/TGFβR1, MAPK, Src, and PI3K to reverse the scattering phenotype without impacting cellular proliferation. Two of these compounds, PD0325901 and saracatinib, enhanced mesenchymal‐epithelial transition (MET) when used in combination in non‐small cell lung cancer (NSCLC) lines (Chua *et al*., 2015). Two other preclinical studies have reported the anti‐EMT activity of Src kinase inhibitors in ovarian and breast carcinoma cell lines (Huang *et al*., 2013; Vultur *et al*., 2008).

A mesenchymal derivative of the HMLE cell model has also been used to identify compounds that promote MET (Pattabiraman *et al*., 2016; Tam *et al*., 2013). In a high‐throughput screen with a firefly reporter linked to the *Cdh1/E‐cadherin*, the authors found that forskolin and cholera toxin effectively induced MET by activating protein kinase A (PKA) through elevating intracellular cyclic AMP. This, in turn, activates PHD finger protein 2 (PHF2), which demethylates histone H3K9me2 and H3K9me3 to derepress epithelial markers and permanently reverse EMT driven by epigenetic mechanisms. Importantly, the resultant MET strongly suppresses the tumor‐initiating capacity and increases the drug sensitivity of EMT‐prone carcinoma lines of various tissue origins. A similar platform also utilized an epithelial marker promoter induction (EpI) screen to identify histone deacetylase inhibitors (HDACi) as a potent class of EMT‐reversing agents (Tang *et al*., 2016; Yun‐Ju Huang and Yo‐Yan Huang, 2016).

An inherent shortcoming of the conventional cell‐based platforms is their inadequacy to model the complex tissue microenvironment in which EMT occurs *in vivo*. To mimic this, a coculturing system employing modern microfluidics has been developed incorporating tumor spheroids in a three‐dimensional hydrogel scaffold (Aref *et al*., 2013). This model also allows for assessing the contribution of endothelial cells in the system. One could expect that, with continual advances in methodology, new facets of the EMT process and, therefore, new strategies of intervention will be uncovered.

Several candidate EMT‐reversing agents are already available clinically, such as saracatinib. Initially developed for the treatment of cancer, saracatinib is a dual‐kinase inhibitor, targeting Src and Bcr‐Abl tyrosine kinases. Although saracatinib is well tolerated in humans and showed promising results in animal studies, its efficacy in clinical trials has been disappointing either alone or in combinatorial treatments (Kim *et al*., 2009; Puls *et al*., 2011). In view of this, the functionally related focal adhesion kinase (FAK) could be tested for EMT reversal properties, as an inhibitor PF‐00562271 has shown encouraging signs in early clinical trials (Infante *et al*., 2012).

A further application of these EMT‐reversing inhibitors would be in combination with other drugs to generate synthetic lethality. Along these lines, small chemical inhibitors of various signaling pathways are currently being used in clinical trials for their anti‐EMT activities. Among these, inhibitors targeting the TGF‐β pathway – a classical activator of EMT – have shown the most promise. Of note, the TGF‐β inhibitor, LY2157299 (galunisertib), is in phase II studies against glioblastoma and hepatocellular carcinoma (Brandes *et al*., 2016; Giannelli *et al*., 2016; Rodon *et al*., 2015). Activation of the AXL receptor is reported to aberrantly phosphorylate SMAD3 to induce EMT in hepatocellular carcinoma (HCC) progression in collaboration with TGF‐β (Reichl *et al*., 2015). As such, the concurrent targeting of AXL and TGF‐β may prove superior to monotherapy aimed at interfering with TGF‐β signaling, and this warrants further investigation, especially given the current availability of AXL inhibitors in the clinic (Antony *et al*., 2016; Byers *et al*., 2013; Feneyrolles *et al*., 2014; Giannelli *et al*., 2016; Nieto, 2013).

Broadly speaking, inhibitors targeting the major cellular signaling pathways often have an impact on the EMT status of the carcinomas, as these pathways are intimately linked with EMT during development (Thiery *et al*., 2009; Voon and Thiery, 2017). It is worth noting, too, the potential hazards of reversing EMT in disseminated tumor cells, as MET is already employed by these metastasized cells as a strategy to promote colonization at distal sites (Beerling *et al*., 2016; Nieto, 2013; Ocana *et al*., 2012; Tsai *et al*., 2012). Therefore, precautions should be observed in the use of EMT‐reversing agents in the clinic and only within a clear therapeutic window.

While these drugs may have anti‐EMT activities, they were developed to target cancer‐driving mutations within these pathways (Table 1). In other words, their clinical benefits are seldom benchmarked against their overall contribution to EMT‐associated tumorigenicity and plasticity. Ironically, their inability to completely inhibit EMT may eventually become a driving force behind chemoresistance against these drugs.

**Table 1:** A list of clinical trials and drug discovery experiments targeting EMT regulatory components

Disease	Tissues	Inhibitors	Targets	Pathway targeted/mechanism	Study type	References
Fibrosis	Kidney	Cyclosporin	Calcineurin	Association of EMT and kidney graft interstitial fibrogenesis	Retrospective	Hazzan *et al*. (2011), Hertig *et al*. (2008)
		Cyclosporin	Calcineurin	Early withdrawal of immunosuppressant did not reduce fibrosis risk in transplant kidneys with EMT features	CERTITEM	Rostaing *et al*. (2015)
Cance	Bladder	Saracatinib	c‐Src	Attenuated growth and metastasis of transplanted tumors	Preclinical	Green *et al*. (2009)
	Breast	SM16	ALK5/TGFβR1	Reducing spontaneous metastases of established allograft tumors	Preclinical	Rausch *et al*. (2009)
		Ki26896	ALK5/TGFβR1	Reduced bone metastasis of breast cancer cell line	Preclinical	Ehata *et al*. (2007)
		1400W, L‐NAME, L‐NMMA	iNOS	Impairment of HIF‐1α and ER stress/TGF‐β/ATF3,4 crosstalk	Preclinical	Granados‐Principal *et al*. (2015)
		EW‐7195/7197/7203, IN‐1130	ALK5/TGFβR1	Inhibition of TGF‐β1‐mediated EMT and metastasis of breast cancer	Preclinical	Park *et al*. (2011a,b), Son *et al*. (2014)
		Salinomycin	LRP6	Identified in high‐throughput screen to show selectivity against CD44^high^/CD24^low^ mammary cancer stem cells	HTS	Gupta *et al*. (2009), Lu *et al*. (2011)
		ML239	NF‐κB pathway	Identified in an expanded screen using the same platform as Gupta *et al*.	HTS	Carmody *et al*. (2012)
	Colon	LY2109761	TGFβRI/II	Reduced liver metastases in a metastatic colorectal xenograft model	Preclinical	Li *et al*. (2010b,b), Zhang *et al*. (2009)
		Sorafenib/regorafenib	SHP1	Activate SHP1 to block TGF‐β‐induced EMT and STAT3 phosphorylation	Preclinical	Fan *et al*. (2015, 2016)
		Emodin	CK2alpha	Inhibition of CK2alpha suppressed tumorigenicity and EMT of CRC cells	Preclinical	Zou *et al*. (2011)
	HNSCC	Gefitinib	EGFR	Gefitinib sensitivity in HNSCC lines is associated with EMT markers	Preclinical	Frederick *et al*. (2007)
		Gefitinib/saracatinib	EGFR/c‐Src	Combined targeting of EGFR and c‐Src effectively inhibited HNSCC growth and invasion	Preclinical	Koppikar *et al*. (2008)
		Cisplatin, cetuximab, and valproic acid	HDAC/EGFR	HDAC inhibitory activity of valproic acid may offer same benefits as vorinostat in suppressing EGFR expression and reversing EMT	Phase II	Bruzzese *et al*. (2011), Caponigro *et al*. (2016)
	HCC	Galunisertib	TGFβRI	Inhibiting TGF‐β signaling restores E‐cadherin expression and diminishes the migratory capacity of HCC cells	Phase II	Giannelli *et al*. (2016, 2014)
		miR‐216a inhibitor	PTEN, SMAD7	miR‐216a/217 targets PTEN and SMAD7 to confer sorafenib resistance	Preclinical	Xia *et al*. (2013)
		miR‐125	SMAD2/4	Interference of SMAD2/4 to attenuate TGF‐β‐mediated chemoresistance	Preclinical	Zhou *et al*. (2015)
	Lung	Erlotinib	EGFR	Erlotinib sensitivity in NSCLC lines and xenografts is determined by EMT status	Preclinical	Thomson *et al*. (2005)
		Erlotinib/PQIP	EGFR/IGF‐1R	EMT status determines the efficacy of combined blockade of EGFR/IGF‐1R in NSCLC lines and xenografts	Preclinical	Buck *et al*. (2008)
		Silmitasertib	CK2	Inhibition of TGF‐β1 induced EMT in A549 cells	Preclinical	Kim and Hwan Kim (2013)
		Silmitasertib	CK2 and FAK–Src–paxillin	Blocks micropillar‐induced FAK activation and EMT	HTS	Kim *et al*. (2015)
		Gefitinib/DN‐30	EGFR/cMET	Concurrent suppression of c‐MET significantly increases gefitinib sensitivity in NSCLC cells	Preclinical	Yano *et al*. (2008), Zucali *et al*. (2008)
		Gefitinib	EGFR	Gefitinib sensitivity of NSCLC lines is correlated with the expression of EMT‐associated markers	Preclinical	Frederick *et al*. (2007)
	Melanoma	PLX4032	BRAFV600E	Significant regression of metastatic melanoma that carries the V600E BRAF mutation	Approved	Flaherty *et al*. (2010)
	Ovary	ABT‐627	ET‐1/ET_A_R‐ILK	Inhibition of ILK suppressed EMT and tumor growth in a xenograft model	Preclinical	Rosano *et al*. (2005)
		ZD4054	ET_A_R/paclitaxel	Cotreatment with ZD4054 sensitized ovarian xenograft tumors to paclitaxel	Preclinical	Rosano *et al*. (2007)
		Saracatinib	c‐Src	Inhibition of c‐Src restored E‐cadherin expression in ovarian cell lines with intermediate mesenchymal state and attenuated spheroid formation	Preclinical	Huang *et al*. (2013)
	Pancreas	LY2109761	TGFβRI/II	Significant reduction in spontaneous abdominal liver metastases in combination with gemcitabine	Preclinical	Melisi *et al*. (2008)

### 2.3 EMT, epigenetics, and chemoresistance

Numerous studies have reported the presence of residual resistant cells following chemotherapy, and these cells have been associated with an EMT phenotype in clinical settings as well as in animal models (Byers *et al*., 2013; Fischer *et al*., 2015; Kitai *et al*., 2016; Manchado *et al*., 2016; Shao *et al*., 2014; Zheng *et al*., 2015). EMT‐associated chemoresistance may also be accompanied with a switch to compensatory pathways, so that carcinoma cells can regain cellular homeostasis (Kitai *et al*., 2016; Manchado *et al*., 2016). While the precise basis for the correlation between EMT and cell survival remains obscure, it is likely that intermediate EMT states offer attractive ‘safe havens’ in which cell signaling can be re‐wired to become independent of the targeted pathway. Here, the capacity to shift to an alternate and viable phenotype relies on the cell's EMT‐endowed plasticity, often termed epithelial–mesenchymal plasticity (EMP) (Byers *et al*., 2013; Nieto, 2013).

It has been proposed that intermediate states represent quasi‐discreet epigenetic states, which are characterized by altered histone modifications on key loci such as *E‐cadherin/Cdh1* and *miR‐200* (Nieto *et al*., 2016; Tam and Weinberg, 2013). Accordingly, the same epigenetic machineries that mark these intermediate states are often implicated in the acquisition of chemoresistance. An important class of such histone modifiers are the polycomb group (PcG) repressor complexes, PRC1 and ‐2. During EMT, the PRC2 complex is recruited to the *CDH1* promoter by the EMT‐TF SNAI1, whereby it catalyzes the trimethylation of histone H3K27 to repress E‐cadherin expression (Herranz *et al*., 2008). The same complex is also responsible for the trimethylation and silencing of miR‐200, which gives rise to chemoresistance (Ceppi *et al*., 2010; Lim *et al*., 2013; Sato *et al*., 2017; Tryndyak *et al*., 2010). PRC1 components, such as BMI1, are considered stem cell factors that support normal stem cells and their transformed counterparts (Park *et al*., 2004; Valk‐Lingbeek *et al*., 2004). The upregulation of BMI1 during carcinogenesis was reported to induce EMT and the invasive phenotype, and this was mediated via its cooperative actions with TWIST1 on *Cdh1* and *INK4A* (Song *et al*., 2009; Yang *et al*., 2010).

Acetylation is another histone modification associated with EMT and chemoresistance. During cancer metastasis, the histone deacetylases (HDAC) 1 and 2 – as part of the Mi‐2–nucleosome remodeling and deacetylase (NuRD) repressive complex – are recruited by Snail and TWIST to the *Cdh1* and *Foxa1* promoters, leading to their repression, respectively (von Burstin *et al*., 2009; Fu *et al*., 2011; Peinado *et al*., 2004; Xu *et al*., 2017). However, various components of the NuRD complex, and specifically the HDACs, will confer drug resistance to cancer cells (Fu *et al*., 2011; Li *et al*., 2014; Sakamoto *et al*., 2016). Consequently, HDAC inhibitors such as vorinostat, mocetinostat, and valproic acid are currently being evaluated as anti‐EMT agents (Bruzzese *et al*., 2011; Caponigro *et al*., 2016; Lan *et al*., 2016; Meidhof *et al*., 2015; Sakamoto *et al*., 2016; Schech *et al*., 2015; Schobert and Biersack, 2017).

A similar correlation between EMT and chemoresistance is also observed for lysine‐specific demethylases, such as LSD1, an emerging class of epigenetic modulators (Bennani‐Baiti, 2012; Lei *et al*., 2015; Nagasawa *et al*., 2015). LSD1 modulates gene expression by removing methyl groups on lysine 4 or lysine 9 of histone H3 to repress or activate target promoters, respectively (Shi *et al*., 2004). In the context of EMT, the induction of EMT in mammary epithelial cells involves the recruitment of LSD1 by SNAI1 to promoters of E‐cadherin, claudin, and cytokeratin family genes, which targets them for repression (Lin *et al*., 2010a,b). In recent years, the association of LSD1 expression with malignancy, chemoresistance, and poor survival has raised interest into the therapeutic potential of its inhibitors (Lv *et al*., 2012; Nagasawa *et al*., 2015; Yu *et al*., 2013b; Zhao *et al*., 2012).

In addition to histone modification, DNA methylation patterns are altered during persistent, mutation‐driven EMT during carcinogenesis (McDonald *et al*., 2011; Tam and Weinberg, 2013). A key mediator of these aberrations appears to be the ten‐eleven translocation 1 (TET1) methylcytosine dioxygenase, which initiates the demethylation of DNA and is associated with tumorigenesis in many cancers (Fu *et al*., 2014; Song *et al*., 2013; Sun *et al*., 2013; Tsai *et al*., 2014). However, there is opposing evidence as to the role of TET1 in EMT‐induced chemoresistance: TET1 has been reported to promote cisplatin resistance through its induction of EMT in ovarian cancer (Han *et al*., 2017), but act as a barrier against EMT in mammary epithelial cells by derepressing the *miR‐200* promoter (Song *et al*., 2013).

Finally, it warrants highlighting that the epigenetic states of the EMT intermediates are cooperatively maintained at multiple levels of epigenetic regulation, with all the usual regulatory elements and limitations of a complex network. For example, just as miR‐200 is a target of PRC2‐mediated repression, the PRC2 component Suz12 is conversely targeted by miR‐200 (Iliopoulos *et al*., 2010; Lim *et al*., 2013). Moreover, a functional crosstalk between TET1 and NuRD during EMT is also likely, given their cooperation in vitamin C‐induced MET during somatic cell reprogramming (Chen *et al*., 2013).

### 2.4 A better mousetrap beyond the EMT spectrum?

From a clinical perspective, the resistance of cancer cells by virtue of their EMT state necessitates targeting the compensatory pathways employed by the cells for their eradication. However, it is just as likely that the very same mechanisms will later give rise to resistance to a new drug. Hence, rather than targeting the ever‐shifting compensatory growth factor pathways, it would seem a better idea to shutdown cellular plasticity. A major obstacle in this approach is that we have an incomplete grasp of the molecular underpinnings of this plasticity. Nevertheless, some cues can be drawn from the field of tissue stem cells, where recent data reveal a genetic program in differentiated cells that promotes cellular plasticity.

Modern lineage tracing studies have demonstrated that some differentiated epithelial cells possess an innate ability to dedifferentiate *in vivo*, and gain multipotency under specific circumstances (van de Moosdijk *et al*., 2017; Rios *et al*., 2016). This phenomenon is most clearly seen during injury and tissue regeneration, but also during inflammation and at certain stages during postnatal development, such as in the mammary gland during pregnancy. Indeed, in specific instances, the induction of stemness is reliant on the coactivation of the EMT program (Guo *et al*., 2012; Ye *et al*., 2015). And, although the precise reason for this association is not known, it is clear that the capacity for somatic cell reprogramming – which was dramatically demonstrated in the generation of induced pluripotent stem cells (iPSc) from terminally differentiated fibroblasts – is integral to tissue homeostasis (van Es *et al*., 2012; Gregorieff *et al*., 2015; Smith *et al*., 2016; Takahashi and Yamanaka, 2006; Tetteh *et al*., 2016). In this light, it is possible that our current investigation of EMT‐associated plasticity and induction would converge on common molecular mechanisms. That is, disease‐associated EMT may be a pathological manifestation of aberrantly activated normal somatic reprogramming of differentiated cells into functional stem cells (Ye *et al*., 2015).

Such a model of common epigenetic pathways governing EMP and induced pluripotency (iP) indeed has the capacity to accommodate common observations between the two phenomena. A prime example of this would be the role of p53 as a barrier, whereby the loss of its function lowers the threshold for entrance into EMP just as it would enhance the iP efficiency (Ansieau *et al*., 2008; Austin *et al*., 2013; Hong *et al*., 2009; Kawamura *et al*., 2009; Marion *et al*., 2009; Mu *et al*., 2017). A significant part of this is mediated through the p53‐miR‐200 regulatory network, which features prominently in the regulation of EMP and iP (Chang *et al*., 2011; Hu *et al*., 2014; Kim *et al*., 2011; Song *et al*., 2013). A further common feature is the repressive effects exerted by lineage‐determining transcription factors, such as BRIGHT/ARID3A, RUNX3, GRHL2, and PAX5 (Chung *et al*., 2016; Hanna *et al*., 2008; Hikichi *et al*., 2013; Popowski *et al*., 2014; Voon *et al*., 2012). Of relevance, both processes are governed by cell extrinsic factors, such as growth factors (van Es *et al*., 2012; Lluis *et al*., 2008; Thiery *et al*., 2009; Vidal *et al*., 2014), and intrinsic epigenetics elements, such as the TET/miR‐200 axis (Hu *et al*., 2014; Song *et al*., 2013) and the NuRD repressor complex (Chen *et al*., 2013; Ebrahimi, 2015; Fu *et al*., 2011; dos Santos *et al*., 2014).

Despite these parallels, there are obvious differences between the induction of EMP in carcinoma and somatic reprogramming, specifically during the generation of iPSc from fibroblasts. Most notably, the induction of pluripotency in the case of the latter is preceded by MET. It reverts fibroblasts into an epithelial phenotype similar to that of embryonic stem cells (Li *et al*., 2010b). Consistent with this, pro‐EMT signals like TGF‐β (Ichida *et al*., 2009; Qin *et al*., 2014; Vidal *et al*., 2014), Wnt/β‐catenin (Ho *et al*., 2013; Lluis *et al*., 2008), and Hippo (Qin *et al*., 2012) pathways act as barriers against iP in a context‐specific manner. At the same time, inhibitors of these pathways, such as the aforementioned anti‐EMT TGF‐β inhibitors, strongly enhance the efficiency of somatic reprogramming (Ichida *et al*., 2009; Maherali and Hochedlinger, 2009). Overall, it seems EMP and iP each require a phenotypic shift along the EMT spectrum (albeit, in opposite directions) toward an intermediate metastable state en route to dedifferentiation and reprogramming. If so, then it is imperative that the innate molecular barriers – such as oxidative and methylation states of the chromatin and their regulators, which safeguard against phenotypic slippage – are thoroughly elucidated. Ultimately, the promise of a plasticity‐centric paradigm is its amenability to the precise targeting of EMT‐associated plasticity in carcinomas irrespective of the upstream driver mutations, and invulnerable to the re‐routing of the signaling circuit observed in current strategies. Accordingly, the development of these next‐generation therapeutics will require discovery platforms that assay the functional output of the involved epigenetic machineries rather than, for example, the activation of a particular marker gene.

## 3 Concluding remarks

EMT has emerged in recent years to be a major driver of chemoresistance to anticancer therapies in the clinic. This is closely linked to phenotypic plasticity in the form of metastable intermediates over the EMT spectrum. The biological reason for this phenomenon is currently unclear, but it is possible that aberrant EMT in carcinoma cells unlocks an innate dedifferentiation program integral to tissue repair, development, and homeostasis. Importantly, such an engine of plasticity would also fuel tumor heterogeneity, progression, and immune escape. Despite the clear need, targeting EMT in cancer therapy has proven challenging due to conceptual difficulties in the design of viable screens. Conventional screening approaches that focus on interfering with specific molecular interactions are unsuitable or have yielded inconsistent results. In this review, we surveyed the current efforts to develop and deploy anti‐EMT therapeutics and discussed their relative effectiveness. By way of this evaluation, a novel concept is put forth to selectively inhibit low‐order epigenetic mechanisms that promote plasticity. In doing so, the phenotypic flexibility that enables cancer cells to be ‘moving targets’ will be greatly restricted, thereby enhancing the efficacies of current therapeutics.
